# Editorial: Women's health in an interdisciplinary dimension – determinants of nutritional disorders

**DOI:** 10.3389/fnut.2024.1492625

**Published:** 2024-09-26

**Authors:** Karolina Krupa-Kotara, Patxi León-Guereño, Izabella Uchmanowicz, Michał Czapla

**Affiliations:** ^1^Department of Epidemiology, Faculty of Public Health in Katowice, Medical University of Silesia in Katowice, Katowice, Poland; ^2^Faculty of Education and Sports, University of Deusto, Bilbao, Spain; ^3^Division of Research Methodology, Department of Nursing, Faculty of Nursing and Midwifery, Wroclaw Medical University, Wrocław, Poland; ^4^Centre for Cardiovascular Health, Edinburgh Napier University, Sighthill Campus, Edinburgh, United Kingdom; ^5^Division of Scientific Research and Innovation in Emergency Medical Service, Department of Emergency Medical, Service, Faculty of Nursing and Midwifery, Wroclaw Medical University, Wrocław, Poland; ^6^Group of Research in Care (GRUPAC), Faculty of Health Sciences, University of La Rioja, Logroño, Spain

**Keywords:** women's health, nutritional disorders, eating disorders, reproductive health, micronutrients

## Introduction

Women's health is a complex and multidimensional issue that requires an interdisciplinary approach that considers both biological and social determinants. In recent decades, there has been a growing interest in the study of women's health, particularly in the context of diet-related diseases. The increase in the prevalence of these diseases in the female population, starting in the second half of the 20th century, can be attributed to increasing life expectancy, as well as increasing exposure to environmental factors, including lifestyle. At the same time, the mechanisms for the development of many diet-related diseases are still incompletely understood, mainly because of their multifactorial etiology.

An important aspect of women's health is understanding how genetic and environmental factors interact to lead to the development of disease. As Judith Stern aptly put it, “*genetics loads the gun, but it's the environment that pulls the trigger*” (1). This means that disease phenotypes are not only the result of interactions between genes but also between genes and environmental factors. In the context of eating disorders in women, this approach is particularly important because a variety of factors, such as diet, physical activity, stress, and cultural differences, can influence health and disease development.

In the analysis of women's health, the role of gender as a variable that differentiates health status cannot be overlooked. Gender is a key determinant of health inequalities, which stem from both biological (sex) and socio-cultural (gender) causes. On the one hand, biological differences affect susceptibility to certain diseases, while on the other hand, social roles, different patterns of health behavior and lifestyles also have a significant impact on women's health. Although women's life expectancy has increased significantly in recent decades, there are still marked differences in health status between different populations, which can be attributed to these very different patterns of social role behavior and cultural factors.

This Research Topic focused on health promotion, disease prevention, and the diagnosis and treatment of all nutritional disorders that affect women's physical social, and emotional wellbeing. The collected research papers, reports, systematic reviews, meta-analyses provide a wide range of perspectives that contribute to understanding the complexity of women's health problems in a global context, consistent with the guidelines of evidence-based medicine and evidence-based nutrition. Among them are papers focusing on the impact of diet and supplementation on women's reproductive health, studies analyzing health and psychological behaviors related to nutrition, and papers addressing specific health problems such as micronutrient deficiencies and the effects of chronic diseases.

**Figure 1:**
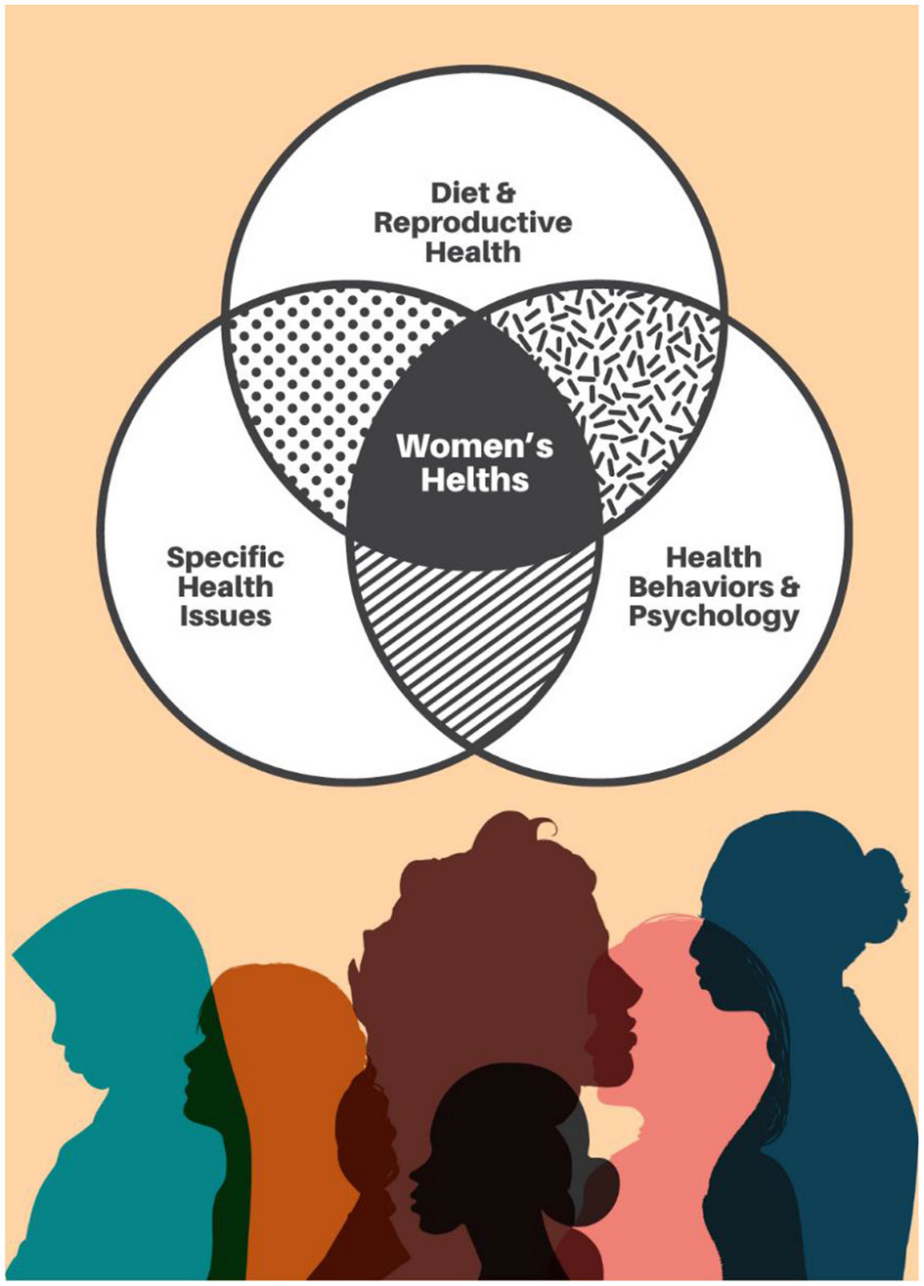
Interdisciplinary intersections in women's health research.

Each of the articles presented brings valuable insights to the interdisciplinary discussion of women's health and eating disorders, addressing a variety of health aspects that are crucial to improving women's quality of life (Figure 1).

## Effects of diet and supplementation on women's reproductive health

One of the first studies on the Research Topic to focus on the effects of diet on women's reproductive health is a paper by Tsushima et al.. This study examines the effects of a ketogenic diet on fertility in patients with polycystic ovary syndrome (PCOS). The results indicate that a ketogenic diet can significantly improve fertility-related parameters, suggesting that dietary changes may be a key element in the treatment of infertility in women with PCOS. Another study by Chen et al. examines the effect of a Mediterranean diet on blastocyst formation in women who have undergone COVID-19 infection. The findings of this study suggest that a Mediterranean diet may promote better blastocyst formation, which underscores the role of a healthy lifestyle and proper nutrition in the treatment of infertility, especially in the context of complications following viral infection.

The next study, conducted by Wang et al., analyzes the relationship between the Dietary Inflammatory Index and infertility in women. The results of this study show that diets with a high inflammatory index, characterized by a high content of processed foods, trans fats and sugars, may contribute to an increased risk of infertility. This study underscores the importance of an anti-inflammatory diet, rich in antioxidants and fiber, in the prevention and treatment of infertility, which is key to improving women's reproductive health.

The Research Topic also includes studies that focus on the impact of diet on maternal and newborn health, emphasizing the importance of proper nutrition during key periods of a woman's life. One such study is the work conducted by Sims et al., which examines the effects of the Mediterranean diet on breast milk composition in women with obesity. The results indicate that a Mediterranean diet, rich in healthy fats, fruits, vegetables, and fish, can positively affect the composition of breast milk. This is particularly important because the quality of breast milk has a direct impact on the health and development of the newborn, as well as on the child's long-term health outcomes. This study underscores the need to promote healthy dietary patterns in lactating women, especially those who are overweight or obese.

The next study, conducted by Alshwaiyat et al., focuses on the effect of diet induced weight loss on iron status and iron indices in young women with overweight or obesity and iron deficiency anemia. This study showed that controlled weight loss through diet can improve iron status in these women, which is crucial for their overall health and reproductive health. The results suggest that dietary interventions aimed at weight reduction should be planned in a way that simultaneously improves nutritional status and prevents micronutrient deficiencies.

The study, by Liu et al., addresses the effect of folic acid supplementation on perinatal mortality in China. The study found that regular folic acid supplementation, especially during the preconceptional and gestational periods, can significantly reduce the risk of perinatal mortality. Folic acid supplementation is widely recommended for the prevention of neural tube defects, but this study also underscores its key role in preventing serious perinatal complications, which is of great public health importance.

A recent study in this group, conducted by Hong et al., focuses on the relationship between maternal mineral status and the risk of preterm birth. The results of this study suggest that mineral deficiencies, including iron and calcium deficiency, may be an important risk factor for preterm birth. This study points to the need to monitor the nutritional status of pregnant women and implement appropriate dietary interventions to prevent negative health consequences for mother and child.

## Health and psychological behaviors related to nutrition

The Research Topic also includes several studies that focus on psychological and behavioral aspects related to nutrition and women's health. Rozmiarek et al. analyze orthorectic behavior among female college students and their motivations for physical activity, eating habits, and restrictive eating behaviors. Orthorexia, or obsession with healthy eating, can lead to serious health disorders, including malnutrition and psychiatric disorders. The results of this study suggest that orthorexia is common among young women, especially in academia, underscoring the need for increased awareness and support for a healthy approach to diet and physical activity.

Another study, conducted by Gogojewicz et al., compared the nutritional status and health habits of women who practice yoga with those who prefer other forms of physical activity. The study found that women who practice yoga tend to have better nutritional status and more balanced eating habits. Yoga, which combines physical and mental elements, can support healthy eating habits and overall wellbeing, suggesting that its regular practice can be beneficial to women's physical and mental health.

The next study, conducted by Mislu et al., focuses on breastfeeding techniques and related factors in northeastern Ethiopia. The results of this study show that proper breastfeeding techniques and maternal education can have a key impact on child and maternal health, especially in terms of improving health indicators and reducing the risk of malnutrition. Breastfeeding education, especially in regions with limited access to health care, is essential for promoting the health and wellbeing of women and their children.

A recent study in this category by Egele and Stark examines how specific health beliefs influence gender differences in dietary choices. The results of the study indicate that men and women differ in their dietary decisions, in part due to different health beliefs. Women, for example, are more likely to choose foods considered “healthier,” such as vegetables and fruits, based on beliefs about their health benefits, while men may prefer protein-rich foods due to beliefs about building muscle mass. This study underscores the importance of incorporating gender differences into health education and dietary interventions to more effectively promote healthy eating habits among both sexes.

## Specific health problems: micronutrients and chronic diseases

Another group of studies on the Research Topic focuses on specific health problems, such as micronutrient deficiencies and the effects of chronic diseases. A study by Hu et al. examines the relationship between micronutrient intake and pelvic inflammatory disease (PID). The results of this study indicate that deficiencies in certain micronutrients, such as zinc and magnesium, may increase the risk of PID, highlighting the importance of adequate nutrition in the prevention of pelvic inflammatory disease. In the context of women's reproductive health, attention to adequate levels of micronutrients in the diet is crucial for the prevention and treatment of such conditions.

The next study, conducted by Sulhariza et al., analyzes changes in hemoglobin levels during pregnancy and their relationship to the risk of gestational diabetes. This study found that a decrease in hemoglobin levels during the first trimester of pregnancy may be associated with a higher risk of developing gestational diabetes. These findings suggest the need for early diagnosis and intervention to improve health outcomes for mothers and their babies, underscoring the importance of monitoring and managing hemoglobin levels during pregnancy.

The study by Białek-Dratwa et al. analyzed dietary trends among Polish women between 2011 and 2022, focusing on the frequency of consumption of different food groups in the Silesia region. The results of this study indicate significant changes in dietary habits that may have a direct impact on population health, including the incidence of diet-related chronic diseases such as obesity, type 2 diabetes, and cardiovascular disease. This study underscores the need for ongoing monitoring of dietary habits in the population and education on healthy eating to counteract the negative health effects of poor eating habits.

A recent study in this group, conducted by Mazza et al., examines the impact of endometriosis on women's dietary choices and daily living activities. Endometriosis, which is a chronic disease that can cause severe pain and fertility disorders, also has a significant impact on women's daily functioning and dietary decisions. The results of this study point to the need for a holistic approach to endometriosis treatment, considering both medical and dietary aspects, to improve patients' quality of life.

## Summary

An interdisciplinary approach to women's health, especially in the context of eating disorders, is essential to understanding the complexity of health problems that affect women at different stages of their lives. The studies and research gathered in this Research Topic highlight how a variety of factors—from genetic to social—affect women's health. Each of the featured articles brings new insights into key aspects of women's health, from diet and lifestyle to psychological and social factors.

These studies show that effective prevention and treatment of eating disorders require an understanding of both the biological and socio-cultural determinants of women's health. Work such as an analysis of the effects of the ketogenic diet on fertility, studies of the inflammatory index of diet vs. infertility, and the role of the Mediterranean diet after COVID-19 provide valuable clues for future health interventions.

In addition, publications on orthorexia, breastfeeding, mineral status in pregnancy, and endometriosis demonstrate the importance of addressing women's specific health needs in the context of their individual experiences and challenges. Ultimately, these studies underscore the need to continue and deepen research on women's health to create more effective health promotion and treatment strategies that address the diverse needs of women around the world.

The research presented here represents an important step toward a more integrated and individualized approach to women's health, considering both the biological and social contexts of health. Findings from this research can contribute to better strategies for preventing and treating eating disorders, ultimately improving the quality of life for women around the world.
